# Overexpression of Melon Tonoplast Sugar Transporter CmTST1 Improved Root Growth under High Sugar Content

**DOI:** 10.3390/ijms21103524

**Published:** 2020-05-15

**Authors:** Baiyi Lu, Suying Wen, Peilu Zhu, Haishun Cao, Yixuan Zhou, Zhilong Bie, Jintao Cheng

**Affiliations:** College of Horticulture and Forestry Sciences, Huazhong Agricultural University and Key Laboratory of Horticultural Plant Biology, Ministry of Education, Wuhan 430070, China; lubaiyii@foxmail.com (B.L.); wensuying@webmail.hzau.edu.cn (S.W.); 18798500269@163.com (P.Z.); chsmugua@webmail.hzau.edu.cn (H.C.); yixuan2000@foxmail.com (Y.Z.); biezhilong@hotmail.com (Z.B.)

**Keywords:** sugar transporter, vacuole, *CmTST1*, melon, root

## Abstract

Sugar allocation is based on the source-to-sink and intracellular transport between different organelles, and sugar transporters are usually involved in these processes. Tonoplast sugar transporters (TST) are responsible for transporting sugar into vacuoles; however, the role of TSTs in root growth and the response to abiotic stress is poorly studied. Here, RNA analysis and promoter-*β*-glucuronidase staining revealed that a melon TST1 gene (*CmTST1*) is highly expressed in the roots. The sugar feeding experiment results showed that the expression of *CmTST1* in the roots was induced by a relatively high level of sucrose (6%), glucose (3%), and fructose (3%). The ectopic overexpression of *CmTST1* in *Arabidopsis* improved the root and shoot growth of seedlings under high exogenous sugar stress. Furthermore, the ectopic expression of *CmTST1* promoted the expression of plasma membrane-located sugar transporters. We proposed that CmTST1 plays a key role in importing sugar transport into the vacuoles of roots in response to metabolic demands to maintain cytosolic sugar homeostasis.

## 1. Introduction

Vacuoles are the largest organelles of plant cells, accounting for more than 90% of the volume of mature cells and containing the largest part of monosaccharides in mesophyll cells [1]. Vacuolar transporters appear to be integrated into a regulatory network that controls plant metabolism [2]. In plant cells, a multitude of sugar transporters are required to regulate the cytosolic sugar concentration [2]. There are three sugar transporter families in plants: monosaccharide transporters (MSTs), sucrose transporters (SUCs/SUTs), and “sugar will eventually be exported transporters” (SWEETs) [2]. The first two families belong to the major facilitator superfamily (MFS), which contains 12 transmembrane domains, whereas SWEETs only contain 7 transmembrane helices, which harbor two MtN3/saliva domains and a loop for connecting [3]. Two subfamilies of MSTs, vacuolar glucose transporters (VGT) and tonoplast sugar transporters (TST), are responsible for importing sugar into the vacuole [4,5,6]. The members of the early responsive to dehydration-6-like (ERD6L) subfamily and the inositol transporter (INT) subfamily of MSTs work as glucose and inositol exporters on the vacuolar membrane, respectively [7,8]. Another type of proton-driven sugar symporters has also been identified in the vacuolar membrane, namely, the sucrose exporter SUC4 [9]. TST and VGT act as vacuolar sugar/proton antiporters, while ERD6L, INT, and SUC4 act as proton/sugar symporters [10]. In addition to these proton-coupled transporters, the tonoplast harbors facilitators that transport their substrates to their respective concentration gradients, such as SWEET 16 [11] and SWEET 17 [12]. These different types of tonoplast-located sugar transporters exert dynamic changes in the vacuolar sugar levels in the different organs of a plant [10].

The root is an indispensable organ for plant growth and development as it plays a role in supporting the plant body, absorbing water and fertilizer, and synthesizing and transferring organic or inorganic substances [13,14]. Sugar can affect plant root growth and development [15]. Sucrose acts as a cotyledon-derived long-distance signal transported by phloem to control root growth [16]. Shoot photosynthesis-derived glucose drives target-of-rapamycin signaling relays through glycolysis and mitochondrial bioenergetics to control the root meristem activation [17]. However, high concentrations of glucose inhibit root meristem growth via ABA INSENSITIVE 5, which represses the PIN1 accumulation and auxin activity [18]. Glucose interacts with auxin signaling and the transport machinery to control a seedling’s root growth and development in changing nutrient conditions [19]. Various glucose concentrations exert not only a change in root length, number of lateral roots, and root hair but also a randomized direction of the roots [19,20].

In our previous research, we found that a melon TST1 gene (*CmTST1*) was highly expressed in the roots [21]. However, the role of CmTST1 in the root system remains unclear. To better understand the function of *CmTST1* in the root and its sugar response, the promoter *β*-glucuronidase (GUS) and coding sequence of *CmTST1* were transformed into *Arabidopsis* separately. The expression of *CmTST1* was detected by quantitative real-time PCR (qRT-PCR) and GUS activity analysis after the seedlings were treated with different compositions and concentrations of sugars. The phenotype of the *CmTST1* overexpression plants was also determined under different sugar treatments. The results revealed that the expression of *CmTST1* can be induced by higher sucrose (Suc), glucose (Glc), and fructose (Fru) levels. The overexpression of *CmTST1* can improve root and shoot growth under high sugar stress. The relationship between *CmTST1* and the sugar signaling pathways will be discussed.

## 2. Results

### 2.1. CmTST1 Is Highly Expressed in the Roots

**Figure 1:**
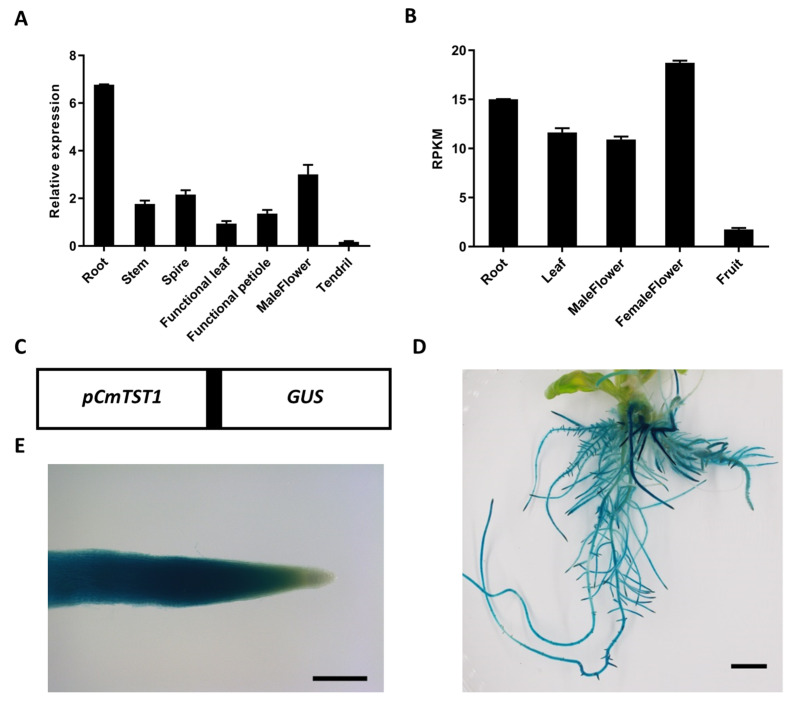
Expression analysis of melon tonoplast sugar transporter gene *CmTST1*. (**A**) qRT-PCR analysis of *CmTST1* in “Elizabeth” melon tissues. (**B**) Expression analysis of *CmTST1* according to the transcriptome data. RPKM, reads per kilobase per million mapped reads; Project, PRJNA383830; Data from cucurbitgenomics (http://cucurbitgenomics.org). (**C**) Diagram of the transcriptional fusion including 2 kb of the 5′ upstream regulatory region of the *TST1* gene (*pTST1*–2 kb) fused to the *GUS* reporter gene that was used to generate the transgenic lines. The corresponding 5′ UTR region is shown as a black box. (**D**) GUS staining in transiently transgenic in melon seedling roots. (**E**) Enlarged root of (**D**). Bar in (**D**) = 1 cm, bar in (**E**) = 250 μm.

In our previous research, we found a strong staining signal in *Arabidopsis* roots expressing the *GUS* gene under the control of the *CmTST1* promoter [21]. To examine the detailed expression profile of *CmTST1* in melon plants, qRT-PCR was performed with RNA extracted from various tissues of 1-month-old plants. The results showed that the expression of *CmTST1* in the root is significantly higher than that in other tissues (Figure 1A). The similar result was showed in transcriptome data (Figure 1B). The melon roots were transiently transformed with the *pCmTST1-GUS* vector through *Agrobacterium rhizogenes*, and the results showed that GUS staining was detected in the whole roots except the root tips (Figure 1C–E). GUS staining in the nontransgenic melon roots as the negative control was shown in supplementary (Figure S1). These results indicated that CmTST1 probably plays an important role in root development.

### 2.2. Expression of CmTST1 Is Induced by Sugar in the Roots

**Figure 2:**
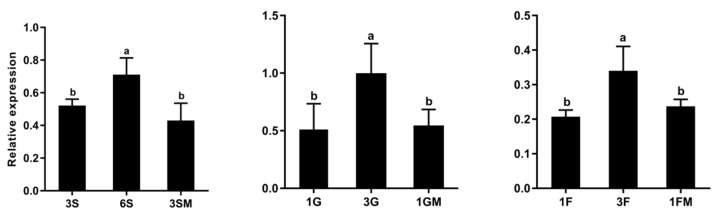
qRT-PCR detected the expression of *CmTST1* in melon roots under different sugar conditions. 3S, 3% Suc; 6S, 6% Suc; 3SM, 3% Suc + 3% Man; 1G, 1% Glu; 3G, 3% Glu; 1GM, 1% Glu + 2% Man; 1F, 1% Fru; 1FM, 1% Fru + 2% Man. Mannitol was added as the control to exclude the influence of osmotic pressure. Lowercased letters above the bar indicate that significant differences between different sugar treatments were assessed by one-sided paired *t*-tests (*p* < 0.05).

Briefly, 6% Suc, 3% Glu, and 3% Fru were set as high sugar levels in contrast to 3% Suc, 1% Glc, and 1% Fru to examine the effects of soluble sugars on the *TST1* gene expression. Mannitol was used as the control to exclude the influence of osmotic pressure. We collected root samples from 1-week-old melon seedlings that were exposed to different sugars to perform the qRT-PCR. The results revealed that the expression level of *TST1* was significantly upregulated in the presence of 6% Suc, 3% Glc, and 3% Fru relative to the levels in the presence of 3% Suc, 1% Glc, and 1% Fru or in the isosmotic control with mannitol (Figure 2).

**Figure 3:**
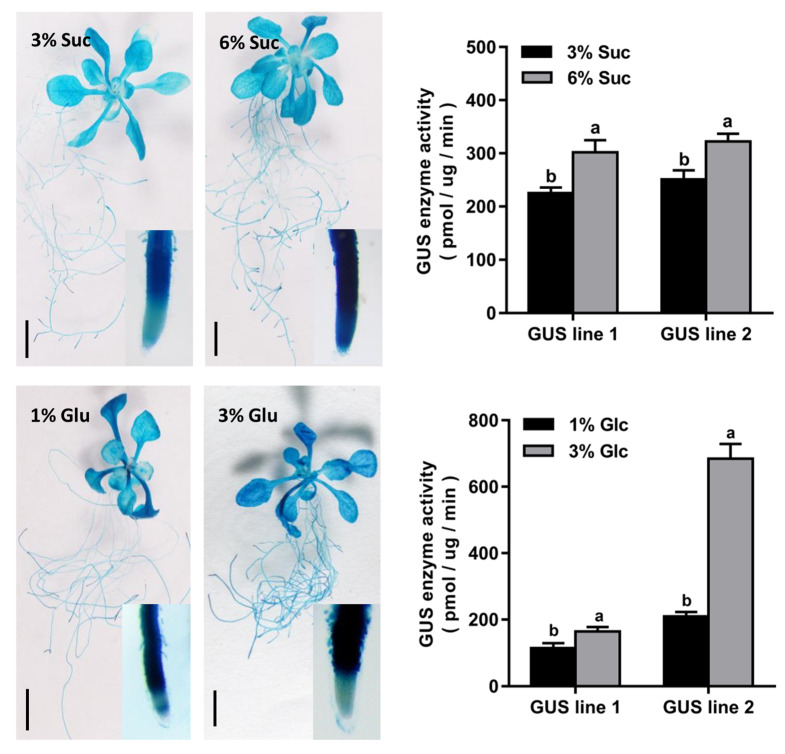

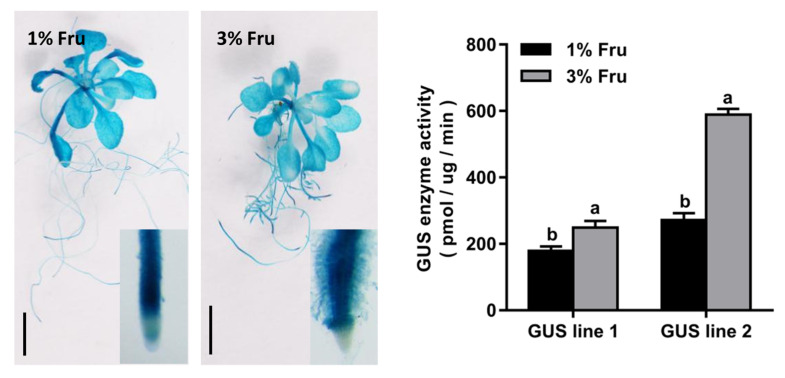
Induction of the *CmTST1*-2K promoter in transgenic *Arabidopsis* in response to different sugars’ supply. Ten-day-old *pCmTST1-GUS Arabidopsis* transgenic plants (T2 generation) were transferred to different sugar conditions (*m/v*) for 7 days. The images on the left show the GUS staining, and the histograms on the right show GUS enzyme activity. Bars = 5 mm. The inserted pictures show the enlarged root. Results represent at least three biological replications. Lowercased letters (a or b) above the bar indicate that significant differences between different sugar treatments were assessed by using one-sided paired *t*-tests (*p* < 0.05).

Furthermore, the GUS expression patterns of the two independent *pCmTST1-GUS* transgenic *Arabidopsis* lines were analyzed in 7-day-old seedlings. After the 7-day treatment, the GUS activity increased in the roots that were exposed to higher Suc, Glc, or Fru levels (Figure 3). The GUS activity was also quantitatively analyzed, and the results indicated that the GUS activity in the high sugar (i.e., 6% Suc, 3% Glc, or 3% Fru) treatments were significantly higher than that in the relatively low sugar (3% Suc, 1% Glc, or 1% Fru) treatments (Figure 3). However, when adding mannitol to compensate for the increased osmotic pressure of the high sugar medium, the expression of *CmTST1* was not affected (Figure S2). These data allowed us to conclusively demonstrate that the accumulation of the *CmTST1* transcript was upregulated by relatively high Suc, Glc, and Fru levels, and this regulation was not related to an osmotic response.

### 2.3. Ectopic Overexpression of CmTST1 in Arabidopsis Improves Root Growth under High Sugar Stress

**Figure 4:**
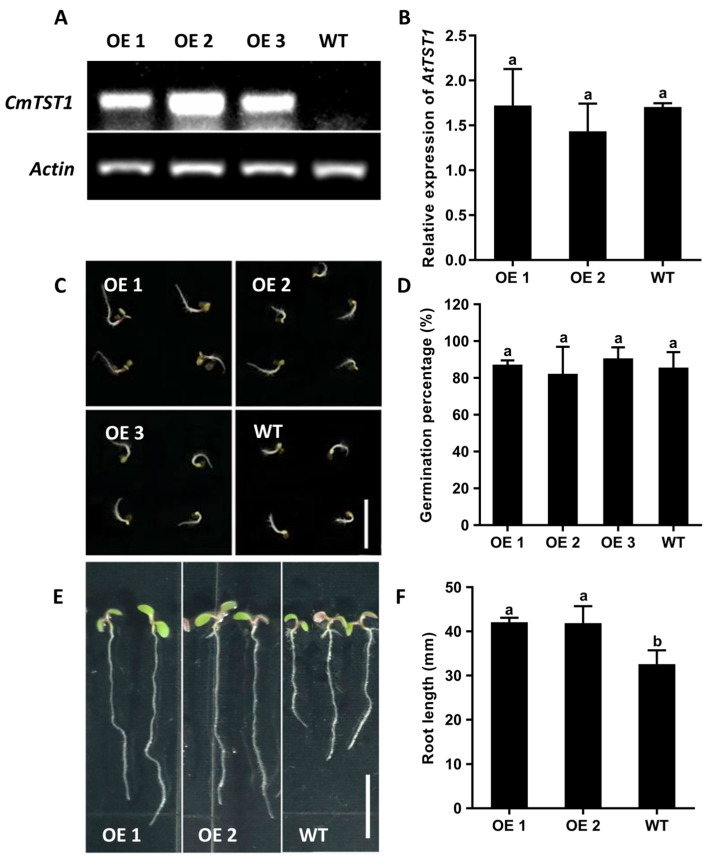
Overexpression of *CmTST1* in *Arabidopsis* seedlings (OE) improved the root growth under a high sucrose level (6%, *w/v*) condition. (**A**) Semi-quantitative PCR analysis of the expression of *CmTST1* in the wild type (WT) and transgenic *Arabidopsis* lines (OE 1, OE 2, and OE 3). (**B**) Comparison of the relative expression of *AtTST1* in OE lines and the WT. (**C**) Phenotype of 3-day-old seedlings grown in 6% Suc. (**D**) Germination percentage of OE and WT *Arabidopsis* seeds. (**E**) Seedlings of OE and WT after growing on 6% Suc for 9 days. (**F**) Average root length of seedlings after growing on 6% Suc for 9 days. WT, wild type. Bar = 5 mm. Results represent at least three biological replications. Lowercased letters above the bar indicate that significant differences between the OE seedlings and the WT were assessed by one-sided paired *t*-tests (*p* < 0.05).

**Figure 5:**
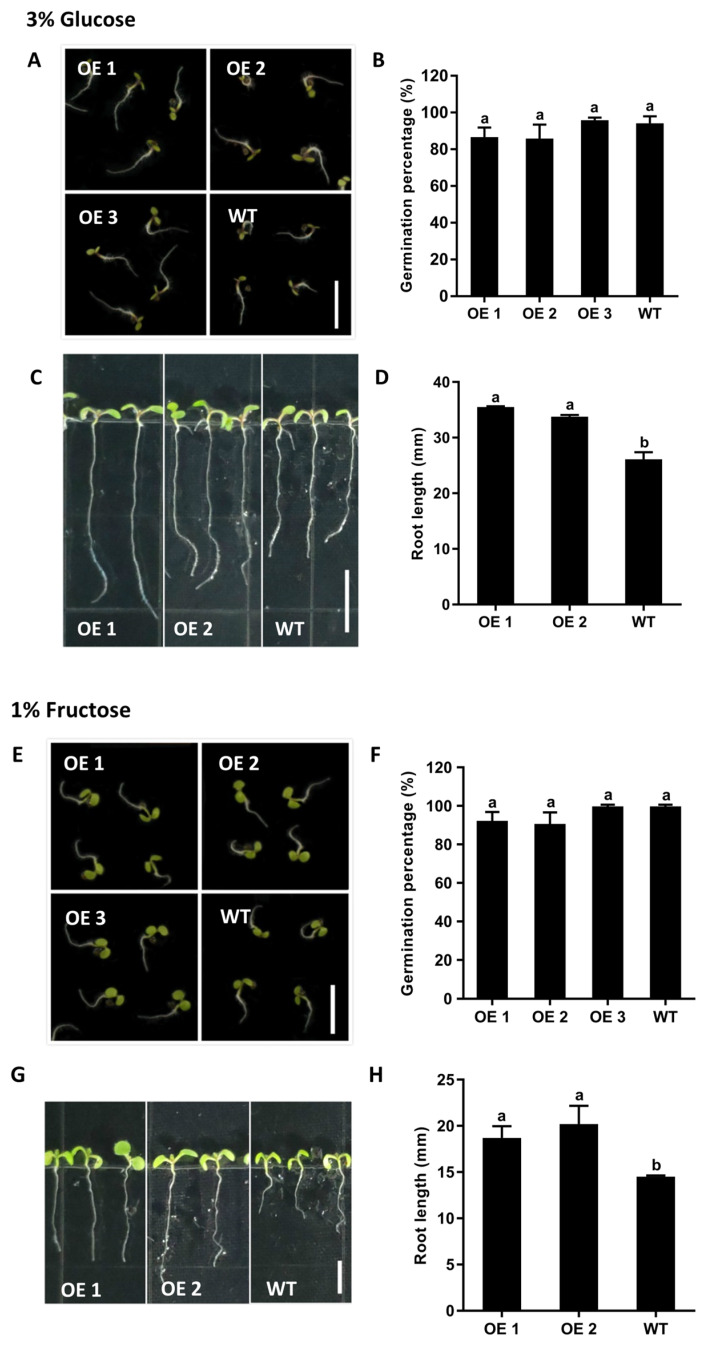
Overexpression of *CmTST1* in *Arabidopsis* seedlings (OE) improved the root growth under (3% *w/v*) Glc and (1% *w/v*) Fru conditions. (**A**,**E**) The phenotype of 3-day-old seedlings. (**B**,**F**) Germination percentage of OE and WT *Arabidopsis* seeds. (**C**,**G**) Seedlings of OE and WT after growing on 3% Glc (C) or 1% Fru (G) 6S for 9 days. (**D**,**H**) Average root length of seedlings after growing on 6S 3% Glc (**D**) or 1% Fru (**H**) for 9 days. WT, wild type. Bar = 5 mm. Results represent at least three biological replications. Lowercased letters above the bar indicate that significant differences between the OE seedlings and the WT were assessed by one-sided paired *t*-tests (*p* < 0.05).

Transgenic *Arabidopsis* plants overexpressing *CmTST1* driven by the Cauliflower mosaic virus (CaMV) 35S promoter were generated to detect the function of CmTST1 in plant root growth. RT-PCR was performed to detect the expression of *CmTST1* in transgenic lines, and the results showed that strong bands can be detected in the *CmTST1* overexpression lines (OE), whereas no signals can be detected in the wild type (WT; Figure 4A). Given that CmTST1 shares a 68.6% amino acid identity with AtTST1 [21], we performed qRT-PCR to detect whether the expression of *AtTST1* was affected in the *CmTST1* OE plants. The results revealed that the *CmTST1* overexpression in *Arabidopsis* did not affect the expression of *AtTST1* (Figure 4B). However, no obvious phenotype was observed when the transgenic lines were planted in a low Suc or Glc source (Figure S3). Considering the high sugar induction expression of *CmTST1*, we planted the T3 generation seeds of the *CmTST1* overexpression plants and the WT seeds in an agar medium with a relatively high sugar content to compare the plant growth. Fifty seeds per treatment were grown on 6% Suc, 3% Glc, or 1% Fru. The OE lines germinated and grew faster than the WT from the pictures taken at the third day after germinating (Figure 4C and Figure 5A,E) However, the germination percentages were calculated five days after germinating, and no difference was observed between the *CmTST1* overexpression lines and the wild type in all three sugar conditions (Figure 4D and Figure 5B,F). After nine days, the roots of the *CmTST1* OE lines were significantly longer than those of the WT in 6% Suc (Figure 4E,F). Similar phenotypes were also observed in 3% Glc and 1% Fru (Figure 5C,D,G,H).

### 2.4. Ectopic Overexpression of CmTST1 in Arabidopsis Affects the Expression of SUC and STP Genes

**Figure 6:**
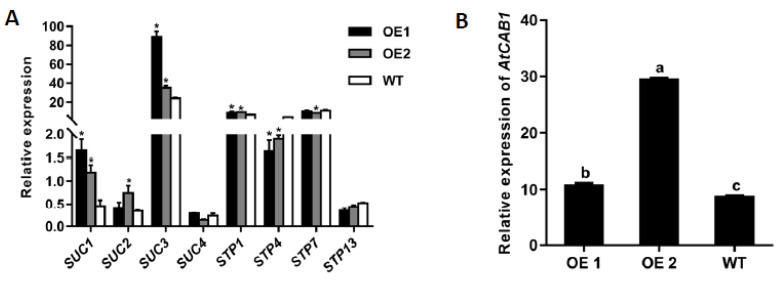
Effect of overexpressing *CmTST1* on the plasma membrane-located sugar transporter expression. (**A**) qRT-PCR detected the expression of *Arabidopsis* sucrose transporter genes (*SUC1*, *SUC2*, *SUC3*, and *SUC4*) and sugar transporter genes (*STP1*, *STP4*, *STP7*, and *STP13*) in the roots of the *CmTST1* OE *Arabidopsis* lines growing under the standard MS medium condition with 3% sucrose (*w/v*) as the only carbon source. (**B**) Comparison of relative expression of *AtCAB1* in the OE lines and the WT. Error bars show the SE of the values from three replicates. * and lowercased letters on the bars indicate a significant difference between the WT and the transgenic lines (*p* < 0.05; Student’s *t*-test).

We determined whether other genes involved in the sugar transport in the *Arabidopsis* root were affected by the overexpression of *CmTST1*. qRT-PCR analysis was used to compare the mRNA accumulation of these genes in the *CmTST1* OE lines or the WT plants grown under 3% Suc and 6% Suc conditions for nine days. Several membrane-located sucrose transporters and sugar transporters were chosen for the analysis. When grown on a 3% Suc medium, the expression levels of *AtSUC1*, *AtSUC2*, *AtSUC3*, and *AtSTP1* in the OE lines were significantly higher than those of the WT; the expression levels of *AtSUC4*, *AtSTP7*, and *AtSTP13* were unchanged, whereas the expression of *AtSTP4* was downregulated (Figure 6A). When grown on a 6% Suc medium, the results showed that the expression levels of *AtSUC1*, *AtSUC2*, *AtSUC3*, *AtSUC4*, *AtSTP1*, and *AtSTP4* in the *CmTST1* OE lines were dramatically upregulated compared with those in the WT. The expression of *AtSTP7* and *AtSTP13* were not affected in the *CmTST1* OE lines (Figure S5). The chlorophyllab-binding protein1 (CAB1) gene was sensitive to glucose in cytosol, and the qRT-PCR results indicated that the expression of *AtCAB1* increased in the *CmTST1* OE lines compared with the WT. (Figure 6B).

## 3. Discussion

### 3.1. Tonoplast Sugar Transporter in the Roots

The tight regulation of sugar compartmentation between the cytosol and vacuole, allowing a dynamic adaptation to altering cellular situations, is important for plant development [10,22]. The roots represent the heterotrophic carbon sink and can be divided into three zones: cell division, elongation, and maturation. Recently, several tonoplast-localized sugar transporters have been identified in the roots. In Arabidopsis, two tonoplast-localization SWEET genes, AtSWEET16 and AtSWEET17, are highly expressed in the root cortex and play a key role in facilitating bidirectional Fru transport across the tonoplast of roots in response to metabolic demands to maintain cytosolic Fru homeostasis [23]. In addition, the expression of the vacuole proton-coupled sucrose exporter gene AtSUC4 is confined to the stele of Arabidopsis roots [9]. Two other types of tonoplast sugar transporter genes, AtINT1 and AtERD6, are also highly expressed in the roots [7,8,24].

TMT/TST genes have been proved to act as a proton-coupled antiporter capable of the high-capacity loading of glucose, fructose, and sucrose into the vacuole [4,25] and play an important role in cellular sugar partitioning and sugar signaling in Arabidopsis [26]. Here, we found that CmTST1 was highly expressed in the roots (Figure 1), which represent heterotrophic carbon sinks and can be divided into zones of cell division, elongation, and maturation. The GUS staining results revealed that the expression of CmTST1 was absent in the root tip meristematic zone cells in most cases (Figure 1E and Figure 3). This is probably because root tip meristematic cells need a large amount of sugar supplied for cell division but not for storage [27], and only small vacuoles are dispersed in the cytoplasm of these cells [28]. In addition, the TST gene has also been detected in particular roots. BvTST2.1 is expressed in the storage cells of sugar beet taproots and is responsible for the sucrose accumulation in sugar beet taproots [6].

### 3.2. Expression of CmTST1 Is Induced by Sugar

Sugars, such as sucrose, glucose, and fructose, not only act as nutritional but also as potent signaling molecules in plants [29]. Sugars regulate the expression of many genes at the transcriptional level in plants, including many sugar transporter genes [30], such as the *Arabidopsis* sugar transporter protein 1 gene, *AtSTP1*, the expression of which is rapidly regulated by the glucose supply [31]. In the present study, we found that the expression of *CmTST1* can be induced by high levels of sucrose, glucose, and fructose (Figure 2 and Figure 3). In *Arabidopsis*, the homologous genes *AtTMT1* and *AtTMT2*, which were first identified as glucose antiporters in the vacuole, respond to high sugar levels [4]. A high glucose level promoted the accumulation of both *AtTMT1* and *AtTMT2* mRNA. However, no obviously increased accumulation in the mRNA of *AtTMT1* and *AtTMT2* could be observed under high-sucrose conditions [4], although *AtTMT1* and *AtTMT2* were proved to transport sucrose in later research [25]. In addition, the expression of *SWEET17*, a facilitative transporter that mediates the fructose transport across the *Arabidopsis* tonoplast, is inducible by fructose in the root [23]. The inducible expression of tonoplast proton-coupled or facilitative sugar transporter genes under high sugar levels is probably because sugar feeding induces carbohydrate accumulation in the cytoplasm and subsequently activates the accumulation in the vacuole. However, the sugar signal that modulates the gene expression takes place in the cytosol and is transmitted through a hexokinase-dependent [32,33] or hexokinase 1-independent signaling pathway [31]. We found many putative sugar responsive cis-acting regulatory elements in the promoter regions of *CmTST1* [21]. However, how the sugar signal modulates the expression of *CmTST1* and regulates plant growth needs further research.

### 3.3. CmTST1 Plays an Important Role in the Root

Plant growth and development require the uptake of soil nutrients by the roots, and root growth is closely associated with sugar concentrations [34]. Sugars are generated from photosynthetic leaves and transported into the roots where they can assist in regulating the nutrient uptake via sugar sensing [35]. Here, we found that *CmTST1* was highly expressed in the root (Figure 1), and that with the sugar sensing pathway as a potential cause, the expression of CmTST1 was induced by a high sugar condition in the media (Figure 2 and Figure 3). To determine the function of *CmTST1* in the roots, we ectopically overexpressed *CmTST1* in *Arabidopsis* and then compared the phenotypes of the *CmTST1* overexpression lines (OE) and the WT when they were planted in different sugar media. No difference could be observed between the OE lines and the WT when grown in an MS medium (containing 3% sucrose) or relatively low glucose conditions (1%) (Figure S3). However, when the sucrose (6%) or glucose (3%) content in the medium was increased, both the roots and shoots of the *CmTST1* overexpression plants grew better than those of the WT (Figure 4, Figure 5 and Figure S4). The *CmTST1* overexpression plants also grew better in the fructose medium (both at 1% and 3%) (Figure 5 and Figure S4). Exogenous glucose or sucrose can promote the growth of plant taproot, however, a high concentration of them can inhibit the growth of taproot and represses the expression of the *CAB1* gene, a sugar-repressed gene [18,36]. The expression of the *CAB1* gene in the WT plant was significantly lower than that in the *CmTST1* overexpression plants after the plant was fed with sugar (Figure 6B). This is because the overexpression of *CmTST1* can reduce the accumulation of sugar in the cytosol by transporting sugar into the vacuole. High glucose and fructose concentrations in the cytosol repress root growth [23,36,37]. The inhibitory effect on root growth could be alleviated by the ectopic overexpression of *CmTST1* (Figure 4 and Figure 5). These results led us to conclude that, physiologically, CmTST1 mediates the glucose, fructose, and probably sucrose uptake into the vacuoles for storage in response to a high concentration of cytosolic sugar.

The overexpression of *CmTST1* also promotes the leaves’ and shoots’ growth under a high sugar condition (Figure S4). However, leaves’ growth is influenced by the root growth status and the leaf photosynthetic capacity. The influence mechanism is complex, so we mainly focus on the function of TST1 in the root system. The function of TST1 in melon leaf and the whole plant need to be further studied in the future.

### 3.4. Overexpression of CmTST1 in Plant Promotes the Expression of Plasma Membrane-Located Sugar Transporters

Sugars are not only energy-rich metabolites providing fuel for cellular machinery, they also constitute key signaling molecules [29]. The uptake of sugars from a medium needs sugar transporters that are located in the membrane. STP proteins and most members of SUC/SUT proteins are usually localized in the plasma membranes [38]. AtSUCs acting as H+/sucrose symporters import sucrose [39]. AtSTPs functioning as H+/glucose and fructose symporters demonstrate an active uptake of glucose and fructose [38,40]. To determine whether the overexpression of *CmTST1* affected the expression of the membrane sugar transporter genes, we chose several members in AtSUCs and AtSTPs to perform the qRT-PCR. The SUC/SUT family was further divided into three subfamilies based on homology: SUC2/SUT1 (contains AtSUC1 and AtSUC2), SUC3/SUT2 (contains AtSUC3), and SUC4 (contains AtSUC4) subfamilies [39]. *AtSUC1*, *AtSUC2*, *AtSUC3*, and *AtSUC4* were selected because they were expressed in the root according to the database (http://bar.utoronto.ca/). *AtSTP1*, *AtSTP4*, *AtSTP7*, and *AtSTP13* were chosen according to the mean expression values averaged from all experiments collected in the Arabidopsis Gene Expression Database (AREX; http://www.arexdb.org). Only *AtSTP1*, *AtSTP4*, *AtSTP7*, and *AtSTP13* showed a significant expression in the roots. The qRT-PCR results showed that the expressions of most of the selected SUC genes increased in the *CmTST1* OE roots when grown in both the 3% Suc and 6% Suc media (Figure 6 and Figure S5). Only one STP gene, *AtSTP1*, was upregulated in the *CmTST1* OE lines under a 3% sucrose condition (Figure 6). The upregulation of AtSUCs and AtSTPs was higher in the 6% Suc than in 3% Suc condition (Figure 6A and Figure S5). The upregulation of AtSUCs and AtSTPs was probably regulated by sugar sensing because the overexpression of *CmTST1* can reduce the sugar content in the cytosol, which stimulates the sugar sensing pathway.

Interestingly, we found that CmSUC3 and CmSUC4 showed a known and putative interaction with CmTST1 by using the STRING website (Figure S6), which is a database of known and predicted protein–protein interactions. These findings suggest an important correlation between TST and SUT proteins. In addition, fructokinase-like 1 (CmFLN1), fructokinase-like 2 (CmFLN2), tocopherol *O*-methyltransferase (CmTOMT), and gibberellin 2-*β*-dioxygenase 2 (CmGA2ox2) were shown to have a putative interaction with CmTST1 (Figure S6).

In conclusion, our results demonstrate that *CmTST1* is highly expressed in the roots of melon in response to high sucrose, glucose, and fructose levels. The ectopic overexpression of *CmTST1* confers tolerance to the inhibition of high sugar levels probably by participating in signaling pathways and affecting the sugar metabolism in *Arabidopsis*.

## 4. Materials and Methods

### 4.1. Plant Materials and Growth Conditions

*Cucumis melo* L. (Elizabeth) were grown in polytunnels from March to June in Wuhan (Midland China). Different tissues (i.e., root, stem, spire, functional leaf, functional petiole, male flower, and tendril) were collected from 1-month-old plants to perform the qRT-PCR analysis. All samples were immediately frozen in liquid nitrogen and stored at −80 °C before analysis.

The *Arabidopsis thaliana* (Columbia) wild type (WT) and transgenic lines were grown on an agar medium in growth chambers at 24 °C and 55% relative humidity under long-day (16 h of light/8 h of dark) conditions.

### 4.2. RNA Isolation, RT-PCR, and qRT-PCR

Total RNA was isolated using TRIzol according to the manufacturer’s instructions (http://www.transgen.com.cn/). The RNA was reversely transcribed using HiScript II Q RT SuperMix for qPCR (+gDNA wiper) (http://www.vazyme.com/). The cDNA was used as a template for the clone, RT-PCR, and qRT-PCR analysis. For the RT-PCR analysis, the gene-specific primers used are listed in Supplementary Table S1. The relative *CmTST1* expression levels were normalized by comparison with *Actin*. qRT-PCR was performed on the ABI7500 system (Bio-Rad, Hercules, CA, USA) using the SYBR green detection protocol (http://www.transgen.com.cn/). Primers used for the qRT-PCR analysis are given in Supplementary Table S1. The relative quantification values for each target gene were calculated by the 2^−ΔΔCt^ method [41] using melon *Tubulin* (CmTub) or *Arabidopsis Actin* (AtAct) as an internal reference gene.

### 4.3. Transient Transformation and GUS Staining in Melon Roots

For the histochemical test of the TST1 spatial expression in melon roots, a fusion expression vector containing a 2 kb fragment upstream from the ATG of CmTST1 and the GUS reporter gene was constructed and named as pCmTST1-GUS (Figure 1C). Based on the hairy root transformation system of melon, pCmTST1-GUS was transformed into melon roots by Agrobacterium rhizogenes (strR, CamR) ArQual Ri (agropine type) (http://www.huayueyang.com) in accordance with the modified protocol of Huang et al. (2019) [42]. Briefly, shelled melon seeds were grown in an MS medium (4.43 g/L MS basal medium with vitamin (G519, www.phytotechlab.com), 30 g/L sucrose, pH 5.6, and 2.5 g/L gellan gum powder (www.phytotechlab.com)) for 1 week before infection. For the sterile growing conditions, melon seeds were sterilized using 0.3% sodium hypochlorite for 15 min and 75% alcohol for 30 s and washed using sterilized water three times. Bacterial colonies were inoculated in a fresh liquid TY medium (5 g/L tryptone, yeast extract 3 g/L; 10 mL/L of 1 M CaCl_2_ was added after sterilization) containing 50 mg/L kanamycin and shaken (220 rpm) at 28 °C overnight until the OD_600_ was 0.6–0.8. The bacterial solution was diluted with a fresh liquid TY medium until the OD_600_ was 0.05 and used to infect the melon seedlings. Sterile melon cotyledons with 1 cm hypocotyl were cut down and immersed into the diluted activated A. rhizogenes solution for 20 min. After infection, the explants were placed onto a filter paper above the co-cultivation medium (4.43 g/L MS, 30 g/L sucrose, pH 5.6, 10 g/L agar, 40 mg/L Acetosyringone) and grown in darkness at 25 °C for 2 days to facilitate a T-DNA transfer from the plasmid into the cells. The explants were subsequently rinsed approximately four times with sterilized water and transferred onto a rooting media (4.43 g/L MS, 30 g/L sucrose, pH 5.6, 2.5 g/L gellan gum powder, 200 mg/mL Timentin). After the rooting media were cultivated in growth chambers for 2 weeks, hairy roots harboring the desired binary vector were identified for the GUS staining. The hairy roots were immersed in a strong staining GUS solution (http://www.obiolab.com/). The air was pumped out for 1 min to make the dye better permeate into the tissues, and the hairy roots were incubated at 37 °C for 12 h. Plants were visualized using stereomicroscopes (M205FA, Leica, Weztlar, Germany).

### 4.4. Sugar Induction Experiment

For the sugar induction expression analysis, two lines of the *pCmTST1-GUS* transgenic *Arabidopsis* reported in our previous research [21] were selected and marked as GUS line 1 and 2. Before the sugar induction treatment, T3 generation seeds of GUS line 1 and 2 were grown in the standard MS (G519, www.phytotechlab.com) solid medium containing 3% (*w/v*) sucrose. *A. thaliana* seeds were sterilized with 0.1% mercuric chloride for 5 min and rinsed four times with water before sowing on agar plates. Approximately 7 days later when two true leaves were growing, seedlings with the same sizes were transferred to different sugar condition (*w/v*) media: 3% sucrose, 6% sucrose, 3% sucrose+3% mannitol, 1% glucose, 3% glucose, 1% glucose + 2% mannitol, 1% fructose, 3% fructose, and 1% fructose + 2% mannitol. Mannitol was used as the negative control to exclude the influence of osmotic stress.

After 7 days of treatment, the seedlings were sampled for the GUS staining or GUS activity analysis. For the GUS staining, the samples were incubated with the GUS staining solution overnight at 37 °C using the method described by Jefferson et al. (1987) [43]. Plants were clarified by using 75% alcohol and visualized using stereomicroscopes (SZX7, Olympus, Beijing, China).

The GUS activity was quantitatively analyzed in accordance with the method of Jefferson et al. (1987) [43]. The fluorogenic reaction was carried out in a 1 mM MUG extraction buffer with a reaction volume of 1 mL. The reaction was incubated at 37 °C, and 200 µL aliquots were removed at 5, 15, 25, 35, and 45 min. The reaction was terminated with the addition of 0.8 mL of 0.2 M Na_2_CO_3_.

### 4.5. Generation of CmTST1 Overexpression Mutants

To construct the CmTST1 overexpression vector, we isolated the coding region of *CmTST1* from Elizabeth melon fruit using PCR and cDNA as a template. The DNA was digested with XbaI/SmaI and cloned into the PBI121 vector under the control of the CaMV 35S promoter. The primers used are listed in Supplementary Table S1. Then, the vector was transformed into Col-0 wild-type *Arabidopsis* plants by using *Agrobacterium tumefaciens* LBA4404 and the floral dip method [44]. For the CmTST1 function analysis, three lines of the T3 generation overexpression mutants were selected and marked as OE 1, OE 2, and OE 3.

The *Arabidopsis CmTST1* OE lines and the WT seeds were sterilized with 0.1% mercuric chloride for 5 min and rinsed four times with water before sowing on agar plates. The media consisted of 4.43 g/L MS basal medium, 2.5 g/L gellan gum powder, and was supplemented with different sugar conditions (*w/v*): 3% sucrose, 6% sucrose, 1% glucose, 3% glucose, 1% fructose, and 3% fructose. To explore the differences between the OE lines and the WT in terms of the germination rate and growth, each plate was divided into four regions, each region was planted with 50 seeds, and each medium had three repeats. The germination rate was calculated after germinating for 5 days, and the growth was observed on the third day. Only 20 seedlings of OE 1, OE 2, and the WT were selected to measure the root length and were measured on the ninth day after germinating. The difference in growth in the above-ground part of the plants of OE 1, OE 2, and the WT was observed with the 30-day-old seedlings.

### 4.6. Bioinformatics Analysis and Statistical Analysis

STRING (https://version11.string-db.org/) is a database of known and predicted protein–protein interactions. The input amino acid sequence of CmTST1 (MELO3C026522) was obtained from CuGenDB (http://cucurbitgenomics.org/).

Student’s t-tests were performed using the algorithm embedded into GraphPad Prism 7, and the significance was evaluated at the 5% level (*p* < 0.05) for all comparisons. For each treatment, the standard error of the mean was calculated on the basis of at least three biological replicates.

## Supplementary Materials

Supplementary materials can be found at https://www.mdpi.com/1422-0067/21/10/3524/s1.
