# Supplementary material for: Overexpression of Melon Tonoplast Sugar Transporter CmTST1 Improved Root Growth under High Sugar Content
Source: Int J Mol Sci. 2020 May 15;21(10):3524. doi: 10.3390/ijms21103524 (PMC7279021; doi:10.3390/ijms21103524)
Supplement: Supplementary file 1 [file ijms-21-03524-s001.zip › ijms-804336-supplementary/Fig.S2.pdf]

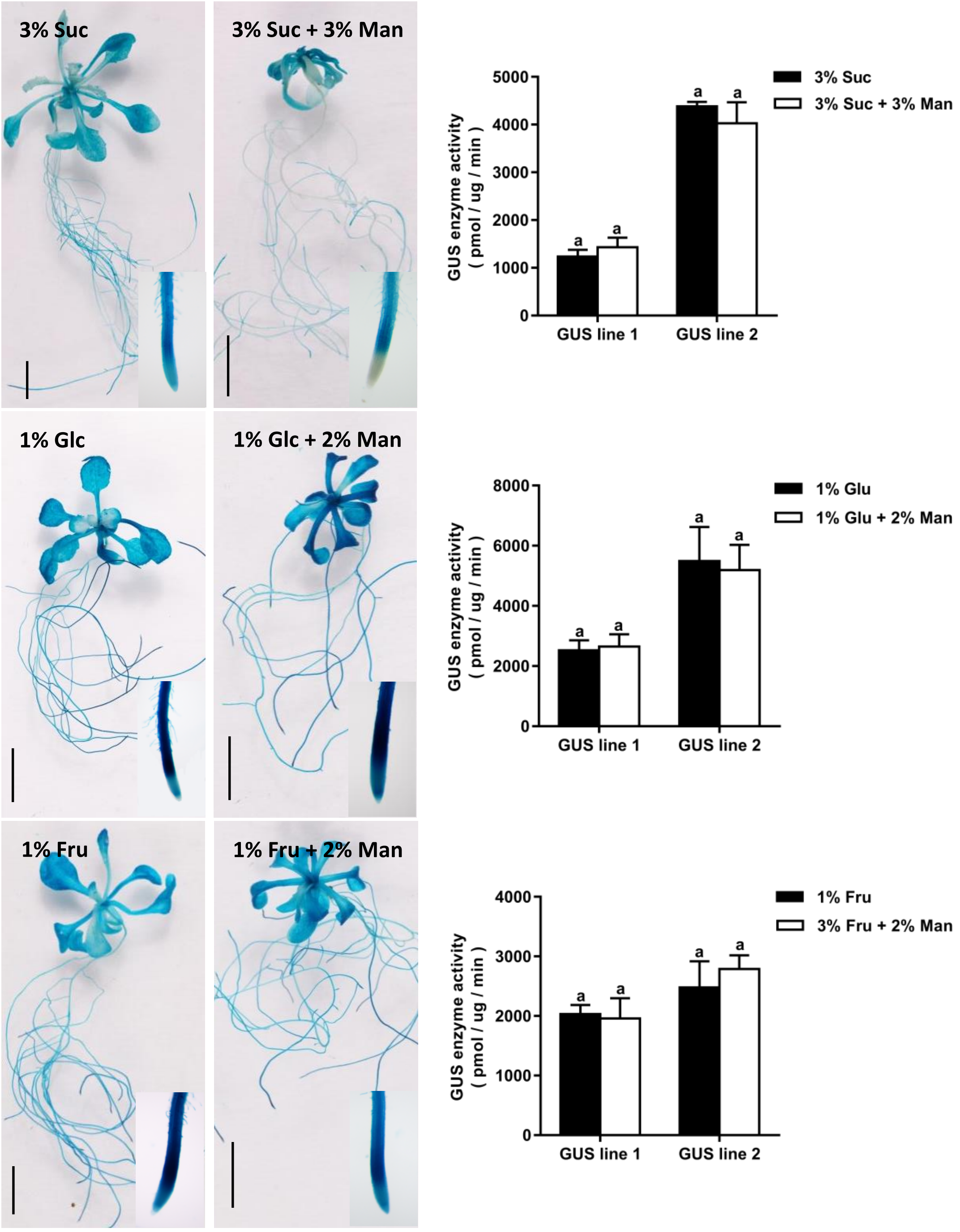

Figure S2. GUS activity analysis of *pCmTST1-GUS Arabidopsis* seedling under mannitol condition to exclude the influence of osmotic pressure for Figure 3. Bars in lower left = 5 mm. The inserted pictures show enlarged root. Bar graph results represent at least three biological replications. Significant differences were assessed by using one-sided paired t-tests ($P < 0.05$). Mannitol was added as the control to exclude the influence of osmotic pressure.
