# Supplementary material for: Overexpression of Melon Tonoplast Sugar Transporter CmTST1 Improved Root Growth under High Sugar Content
Source: Int J Mol Sci. 2020 May 15;21(10):3524. doi: 10.3390/ijms21103524 (PMC7279021; doi:10.3390/ijms21103524)
Supplement: Supplementary file 1 [file ijms-21-03524-s001.zip › ijms-804336-supplementary/Fig.S4.pdf]

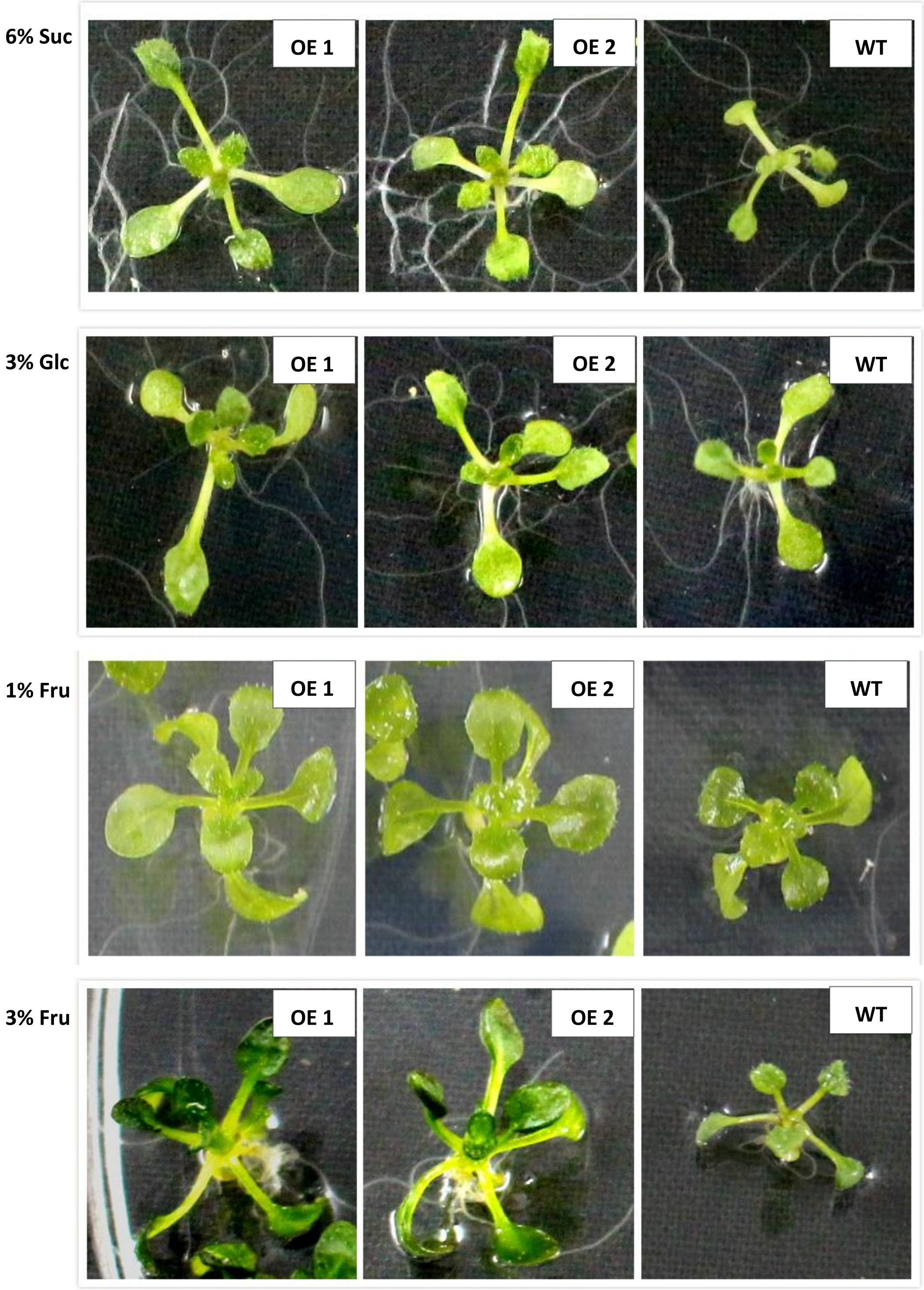

Figure S4. Thirty-day-old WT and *CmTST1*-OE seedlings grown on MS media supplemented with 6% sucrose, 3% glucose, 1% fructose, or 3% fructose. OE, *CmTST1* overexpression *Arabidopsis*, WT, wild type.
