# Supplementary material for: Overexpression of Melon Tonoplast Sugar Transporter CmTST1 Improved Root Growth under High Sugar Content
Source: Int J Mol Sci. 2020 May 15;21(10):3524. doi: 10.3390/ijms21103524 (PMC7279021; doi:10.3390/ijms21103524)
Supplement: Supplementary file 1 [file ijms-21-03524-s001.zip › ijms-804336-supplementary/Fig.S5.pdf]

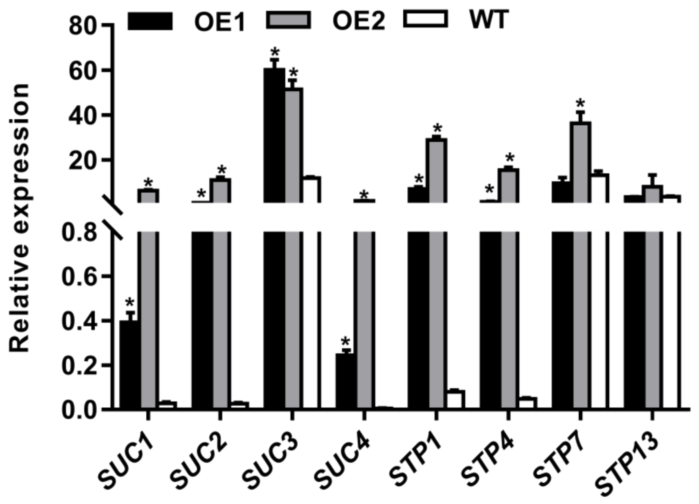

Figure S5. qRT-PCR detected the expression of *Arabidopsis* sucrose transporter genes (*SUC1*, *SUC2*, *SUC3*, and *SUC4*) and sugar transporter genes (*STP1*, *STP4*, *STP7*, and *STP13*) in the roots of *CmTST1*-OE *Arabidopsis* lines growing under 6% Suc condition. Error bars show the SE of the values from three replicates. * on the bars indicates significant difference between WT and transgenic lines ($P < 0.05$; Student's t-test).
