# Supplementary material for: Overexpression of Melon Tonoplast Sugar Transporter CmTST1 Improved Root Growth under High Sugar Content
Source: Int J Mol Sci. 2020 May 15;21(10):3524. doi: 10.3390/ijms21103524 (PMC7279021; doi:10.3390/ijms21103524)
Supplement: Supplementary file 1 [file ijms-21-03524-s001.zip › ijms-804336-supplementary/Fig.S6.pdf]

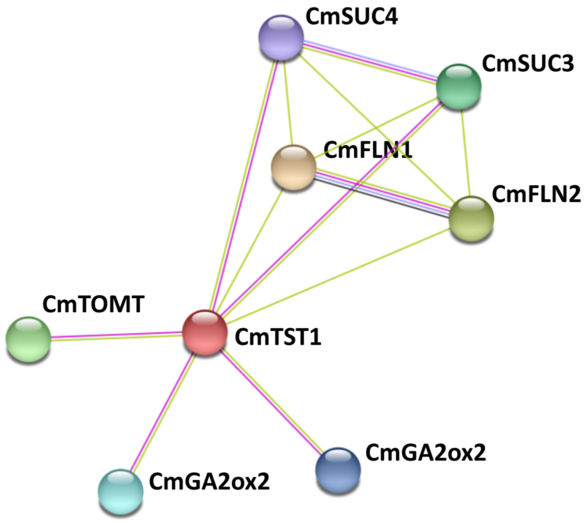

Figure S6. A prediction of protein–protein interactions of CmTST1 STRING (https://version11.string-db.org/) Network nodes represent proteins. All proteins were from *Cucumis melo* organism. Red node represents CmTST1 (XP_008464819.1), tonoplast sugar transporter. Orange node represents CmFLN1 (XP_008442480.1), fructokinase-like 1. Yellow node represents CmFLN2 (XP_008442238.1), fructokinase-like 2. Purple node represents CmSUC4 (XP_008444969.1), sucrose transport protein SUC4 isoform X1. Dark green node represents CmSUC3 (XP_008465743.1), sucrose transport protein SUC3. Light green node represents CmTOMT (XP_008462787.1), tocopherol *O*-methyltransferase. Light blue node represents CmGA2ox2 (XP_008461412.1), gibberellin 2-β-dioxygenase 2. Deep blue node represents CmGA2ox2 (XP_008447463.1), gibberellin 2-β-dioxygenase 2. Edges represent protein–protein associations. Purple edge shows known interaction that was determined experimentally. Yellow edge represents the textmining interaction. Blue edge represents for predicted interaction of gene co-occurrence. Black edge shows the co-expression.
