# Supplementary material for: Overexpression of Melon Tonoplast Sugar Transporter CmTST1 Improved Root Growth under High Sugar Content
Source: Int J Mol Sci. 2020 May 15;21(10):3524. doi: 10.3390/ijms21103524 (PMC7279021; doi:10.3390/ijms21103524)
Supplement: Supplementary file 1 [file ijms-21-03524-s001.zip › ijms-804336-supplementary/Table. S1.pdf]

**Table S1.** Primers used in the paper (lowercased letters indicate the restriction enzyme sites or recombination connector sequence).

| *Primers* | *Description* | *Sequence (5'- -3')* |
|---|---|---|
| CmTST1-121F | clone in to PBI121 | cacggggactctagaggatcc ATGAAGGGAGCTTTGCTAGTGGC |
| CmTST1-121R | clone in to PBI121 | ggactgaccacccggggatcc TCAATTAATACTACCTTTCGCAGCT |
| TST1PF | Promoter clone | gaccatgattacgccaagcttGTCATATACTTACTTTAGCTCAACCTC |
| TST1PR | Promoter clone | ggactgaccacccggggatccTTATTCAATTCAAGCTCCAATGTGGTCAG |
| Cm18SF | Semi RT-PCR | CGAGTCTGGTAATTGGAATGAGTA |
| Cm18SR | Semi RT-PCR | CTACGAGCTTTTTAACTGCAACAA |
| TST1F | Semi RT-PCR | GCATCAGAGGCTGGTGGAG |
| TST1R | Semi RT-PCR | TAGCACAGGGATGGTAGCG |
| CmTubF | Quantitative RT-PCR | AACTCCACCAGTGTAGCCG |
| CmTubR | Quantitative RT-PCR | CACCCACCTCTTCATAATCC |
| CmTST1qF | Quantitative RT-PCR | ATAATAGGTCCTGCTGAAGA |
| CmTST1qR | Quantitative RT-PCR | AAAGGGTGACAAGTGGGT |
| AtActqF | Quantitative RT-PCR | CTTGCACCAAGCAGCATGAA |
| AtActqR | Quantitative RT-PCR | CCGATCCAGACACTGTACTTCCTT |
| AtTST1qF | Quantitative RT-PCR | GAATGGACGAGGCTAAGCGA |
| AtTST1qR | Quantitative RT-PCR | TGGGTTCCATAAAGCCGCAT |
| AtSTP1qF | Quantitative RT-PCR | GGGGACGTCGGTTTCTCTTT |
| AtSTP1qR | Quantitative RT-PCR | CCGACCTTATCTCCAACGGG |
| AtSTP4qF | Quantitative RT-PCR | AGGAGCTTTCAACAACGGGT |
| AtSTP4qR | Quantitative RT-PCR | GCCACGTTCGATGAGAGAGT |
| AtSTP7qF | Quantitative RT-PCR | GGTCATGGGGTCCTCTAGGT |
| AtSTP7qR | Quantitative RT-PCR | ACTGTCACCCAACCAGCAAA |
| AtSTP13qF | Quantitative RT-PCR | TCCTCTCTATGCTTTGCCATTTC |
| AtSTP13qR | Quantitative RT-PCR | AGTCTCCGGAAGTAGGAACATCAC |
| AtSUC1qF | Quantitative RT-PCR | ACACAGTCGCCGGAAGATTT |
| AtSUC1qR | Quantitative RT-PCR | AGAGAGAGCTGTAAGGCCCA |
| AtSUC2qF | Quantitative RT-PCR | CATCCTCGCCATTTGCTTGG |
| AtSUC2qR | Quantitative RT-PCR | AGCTGTGACGTTACCAGGTG |
| AtSUC3qF | Quantitative RT-PCR | GGATTTTCTGCAGACATTGGGT |
| AtSUC3qR | Quantitative RT-PCR | AGCCATCCACAAGCAGAACA |
| AtSUC4qF | Quantitative RT-PCR | TCACAGAGTCACTCGCAACC |
| AtSUC4qR | Quantitative RT-PCR | ACCGCAAAGCCAAATCACAC |
| AtCAB1-qF | Quantitative RT-PCR | GCCGGTAAGGCCGTC |
| AtCAB1-qR | Quantitative RT-PCR | GGTGTCCCATCCGTAGTCTC |
